# Re-Evaluating the Internal Phylogenetic Relationships of Collembola by Means of Mitogenome Data

**DOI:** 10.3390/genes12010044

**Published:** 2020-12-30

**Authors:** Claudio Cucini, Pietro P. Fanciulli, Francesco Frati, Peter Convey, Francesco Nardi, Antonio Carapelli

**Affiliations:** 1 Department of Life Sciences, University of Siena, Via A. Moro 2, 53100 Siena, Italy; claudio.cucini@student.unisi.it (C.C.); paolo.fanciulli@unisi.it (P.P.F.); francesco.frati@unisi.it (F.F.); francesco.nardi@unisi.it (F.N.); 2 British Antarctic Survey, NERC, High Cross, Madingley Road, Cambridge CB3 0ET, UK; pcon@bas.ac.uk

**Keywords:** springtail, evolution, mitochondrial DNA, phylogeny

## Abstract

Collembola are an ancient and early diverging lineage of basal hexapods that occur in virtually all terrestrial habitats on Earth. Phylogenetic relationships between the different orders of Collembola are fiercely debated. Despite a range of studies and the application of both morphological and genetic approaches (singly or in combination) to assess the evolutionary relationships of major lineages in the group, no consensus has been reached. Several mitogenome sequences have been published for key taxa of the class (and their number is increasing rapidly). Here, we describe two new Antarctic Collembola mitogenomes and compare all complete or semi-complete springtail mitogenome sequences available on GenBank in terms of both gene order and DNA sequence analyses in a genome evolution and molecular phylogenetic framework. With minor exceptions, we confirm the monophyly of Poduromorpha and Symphypleona *sensu stricto* (the latter placed at the most basal position in the springtail phylogenetic tree), whereas monophyly of Neelipleona and Entomobryomorpha is only supported when a handful of critical taxa in these two lineages are excluded. Finally, we review gene order models observed in the class, as well as the overall mitochondrial nucleotide composition.

## 1. Introduction

Collembola (springtails) are the largest and most diverse group amongst basal hexapods, comprising over 7000 species worldwide and inhabiting almost every terrestrial habitat on Earth, from ice-free areas of the polar regions to tropical deserts. Soil, litter, and ground vegetation are their natural habitats, where they play a pivotal role in the decomposition of organic matter [1]. Their ancient evolutionary origin was dated as early as the Silurian (443–419 million years ago), suggesting that their diversification into several major lineages may have taken place in parallel with the evolution of vascular plants and the processes of soil formation [2]. Apart from the never-questioned monophyly of the class, the phylogenetic relationships of Collembola in the context of the Arthropoda have been a long-debated topic, with a range of alternative hypotheses emerging in the last 20 years [3,4,5]. 

**Figure 1:**
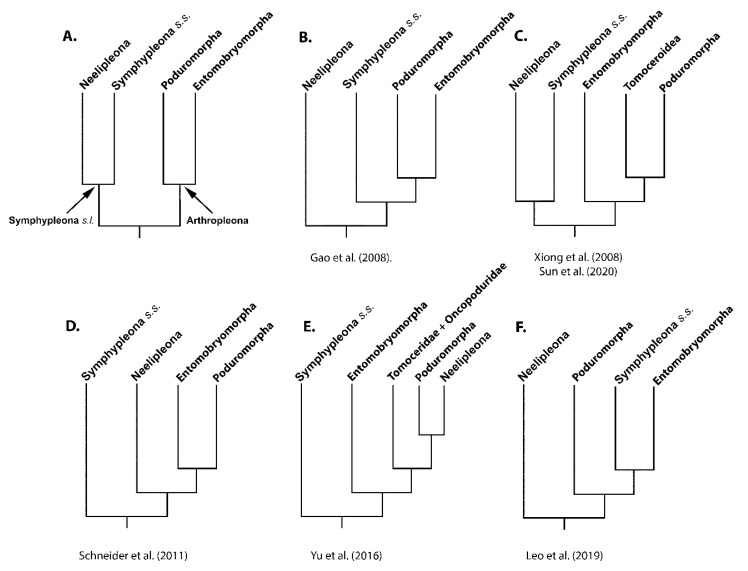
Competing hypotheses for Collembola phylogeny obtained using molecular markers and listed in chronological order. (**A**) Traditional view; (**B**) Based on ribosomal markers [9]; (**C**) Based on ribosomal [11] and mtDNA protein coding genes [14]; (**D**) Based on *16S*, *cox1* and *28S* markers [10]; (**E**) Based on ribosomal markers [12]; (**F**) Based on mitochondrial protein coding genes [2].

In the early days of Collembola systematics, different lineages were classified based on gross body morphology and, later, based on the analysis of shared morphological characters in a cladistic framework (see [6] for a complete review). Based on these approaches, Collembola were subdivided into two suborders: Arthropleona (with an elongated body and well-defined segments) and Symphypleona *sensu lato* (with a globular body shape and fused body segments) [1] (Figure 1A).

Early millennial morphological data [6] already started to cast doubt on this traditional Collembola systematic framework and, with the progressive application of molecular techniques, progress was made towards resolving the group’s debated internal relationships [4,7], leading to the acceptance of four main orders and highlighting the enigmatic taxonomic position of only a few groups [8]. The Arthropleona were subdivided into Entomobryomorpha and Poduromorpha, while Symphypleona *s.l.* was subdivided into Symphypleona *sensu stricto* and Neelipleona [6,8] (Figure 1A). Subsequently, inter-class relationships have become the new subject of debate amongst systematists, with at least five competing hypotheses proposed, and nuclear and/or mitochondrial DNA markers increasingly becoming the main source of data.

The first complete phylogenetic survey based on *18S* and *28S* rDNA sequences was that of [9]. Neelipleona was retrieved as basal to Symphypleona *s.s.* (hereafter Symphypleona) and a Poduromorpha + Entomobryomorpha clade (Figure 1B). Other authors [10], based on the study of three different loci (*16S*, *cox1* and *28S*), developed a similar phylogenetic hypothesis, within which the positions of Symphypleona and Neelipleona were inverted (Figure 1D). While including representatives from all classes, these analyses did not include problematic taxa, such as Tomoceridae and Oncopoduridae, whose phylogenetic relationships were not assessed on morphological grounds. These latter were included in the analyses of [11], who proposed that Symphypleona + Neelipleona were a sister group to an Entomobryomorpha + (Tomoceroidea + Poduromorpha) clade, confirming a previous result obtained with an incomplete data set [8] (Figure 1C). Based on analyses of a larger data set and the use of two nuclear rDNA genes as markers, a further phylogenetic arrangement has been proposed where Symphypleona figured as the sister taxon to Entomobryomorpha (partial) + (Tomoceridae + Oncopoduridae + (Poduromorpha + Neelipleona)) [12] (Figure 1E).

As the costs of next generation sequencing (NGS) reduce and access to the technique becomes more widespread, along with the development of improved protocols for non-model species, sequence data are accumulating at an unprecedented rate. Nuclear genomic data have been used to unravel deep-level phylogenetic relationships in hexapods, including early diverging lineages [13], but the limited number of representatives for each major group does not currently allow a thorough investigation of internal relationships at lower taxonomic levels. In this context, complete mitochondrial genomes appear to be a suitable marker, and their informativeness in terms of infraorder relationships among springtails has been assessed in certain studies [2,14,15]. In consequence of the factors of uniparental inheritance, lack of recombination, haploidy, technical feasibility of sequencing using NGS technologies and occurrence in almost all Eukaryota, complete mitochondrial genomes have now emerged as a very popular molecular marker in phylogenetic studies. Moreover, the conservation of genes in the mitogenome minimizes the possibility that paralogous genes are sampled, and the order of genes along the molecule (gene order) provides an independent set of characters that can complement sequence analysis [16,17,18]. Different sets of complete mitogenomic data have been applied in the study of internal relationships among Collembola [19]. Leo and colleagues [2] proposed Neelipleona as the earliest diverging lineage in Collembola, although not all analyses converged to the same result, while Poduromorpha was recovered as the sister taxon to the more derived Entomobryomorpha + Symphypleona (Figure 1F). Sun and colleagues [14], in turn, support a scenario similar to that proposed by [11] (Figure 1C). A third relevant study [15], focusing on internal relationships among species of a single genus, did not address inter-ordinal relationships but provided a wealth of primary data. With these three studies appearing independently but almost at the same time, and with additional genomes previously available on GenBank, this meant that different non-overlapping sets of species were included. A global re-analysis of all available complete springtail mitochondrial genomes available at present is necessary.

**Figure 2:**
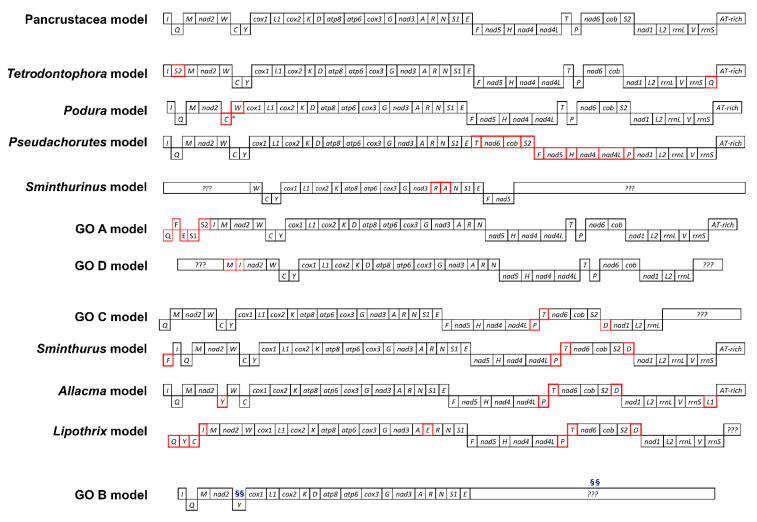
Collembola gene orders available up to June 2020. The Pancrustacean model is considered to be ancestral within the springtail lineage. All derived gene arrangements, with respect to the basal model, show translocations as red boxes, deletions as red asterisks (*), and possible translocations as blue section signs (§). Incomplete sequences are identified with question marks.

Alongside sequence analysis, gene order data have emerged as a further informative phylogenetic marker. Despite the fact that gene order mutation mechanisms remain a matter of discussion (see [18] for a complete review), the sharing of a derived gene order is regarded as a very strong signal of shared ancestry due to the rarity of rearrangements and the low chance of observing convergent changes. To date, twelve different gene order models have been described among Collembola, although four (GO A, GO B, GO C, GO D, revised in a later section) are derived from a metagenomic survey and therefore their species of origin is not identifiable (Figure 2) [2,20]. It is now widely accepted that the most frequent gene order in springtails is the Pancrustacea model. This arrangement, in turn the most common in hexapods and crustaceans, is frequently observed in all springtail lineages and is regarded as the ancestral state for the class [5,21]. With minor exceptions, it characterizes the Entomobryomorpha and Neelipleona. The second most common gene order is the *Tetrodontophora* model, present in all species of the family Onychiuridae investigated to date, which shows a translocation of two tRNA-encoding genes (*trnSuga* and *trnQ*) compared to the ancestral gene order. Among Poduromorpha, two other gene arrangements have been detected, the *Podura* and *Pseudachorutes* models, as observed in *Podura aquatica* [22] and *Pseudachorutes palminensis* [23], respectively. The former model is characterized by a double translocation (*trnC*, *trnW*) plus a gene loss or translocation of *trnY* (the mtDNA is partially incomplete and the position of this gene could not be assessed) with respect to the Pancrustacea model. The *Pseudachorutes* model displays the first (and unique, to date, among springtails) translocation which also involves some protein coding genes. Within Symphypleona, rearrangements are observed at high frequency in the family Sminthuridae, regarded as a “hot spot” for genetic rearrangements. All species of this group investigated to date display rearranged genomes (Figure 2). The *Sminthurinus* model, only detected in *Sminthurinus signatus*, shows an inversion between *trnA* and *trnR*. GO A and GO D, observed in some unidentified Symphypleona species, show different rearrangements. The former displays multiple gene translocations, namely *trnQ-trnF-trnE-trnS1-trnS2* between the AT-rich region and *trnI* combined with inversion of the block *trnF-trnE-trnS1*. GO D, even though gene annotation along the sequence was not complete, shows a reciprocal exchange between *trnM* and *trnI*. It was proposed that a *trnP* and *trnT* inversion plus a *trnD* translocation were a common feature of the Sminthuridae ancestor and, hence, inherited by all its descendent lineages [24]. Four different gene orders in this taxon have been described: the GO C model, which only shows the putative Sminthuridae ancestral translocations; the *Sminthurus* model, which shows a *trnF* translocation; the *Allacma* model consisting of a *trnY* and a *trnLuaa* translocations; and the *Lipothrix* model, which displays four translocations (*trnQ*, *trnY*, *trnC* and *trnI*). The twelfth gene order described to date, GO B, belongs to an unknown species of Lepydocyrtidae whose mtDNA was only partially sequenced. This, with the translocation of *trnW* and *trnC* to unknown positions, apparently represents the only genetic rearrangement found within Entomobryomorpha.

In the light of the considerable interest in the mitochondrial genome as a potential marker to establish infraorder phylogenetic relationships, and considering that all available complete mitochondrial genomes have never been included in a single study, we here revise all available mitogenomic information for Collembola and provide a phylogenetic analysis, gene order analysis and base frequency bias analysis for all known sequences in a unitary framework. We further describe and analyze the newly determined mitogenomes of two Antarctic springtails: *Kaylathalia klovstadi* (firstly described as *Isotoma klovstadi* [25], then provisionally re-assigned to *Desoria klovstadi* [26] and revised by [27]) and *Tullbergia mixta*.

## 2. Materials and Methods 

### 2.1. Sample Collection and Mitochondrial DNA Sequencing

Specimens of *T. mixta* were collected in January 2003 at Harmony Point, Nelson Island (South Shetland Islands; 62° 13′ 20.7′′ S 58° 46′ 54.0′′ W) during the 2002/2003 field season of the British Antarctic Survey (BAS). Specimens of *K. klovstadi* were collected in January 2019 at Redcastle Ridge (Continental Antarctica; 72° 26′ 25.0′′ S 169° 56′ 32.0′′ E) during the Italian National Antarctic Program (PNRA) expedition. Total DNA was extracted using the QIAmp^®^ UCP DNA kit from pools of 10–15 individuals of each species. Extractions were then normalized, pooled, and sequenced at Macrogen Europe using a TruSeq Nano DNA chemistry on a NovaSeq 6000 platform (Illumina, San Diego, CA, USA), together with additional samples not reported in this study, to produce 151 bp paired end reads.

### 2.2. Read Assembly and Mitogenome Annotations

Resulting reads were quality controlled using FastQC (ver. 0.11.9; available at https://www.bioinformatics.babraham.ac.uk/projects/fastqc/) and then assembled using NOVOPlasty v.3.8.3 [28]. The assembly step was carried out using the four available *cox1* sequences for *K. klovstadi* downloaded from the Bold System (AN: GBCO0087/88/89/90-06), and the *T. mixta* partial sequence downloaded from GenBank (AN: KF982833) as seeds. Initial assemblies were obtained under default settings. These were in turn used as a filter to create a subset of the reads enriched for mitochondrial sequences using the filter_reads.pl script from the same package. In turn, final assemblies were produced, based on this enriched library, using the full data. Resulting contigs were compared to MEGAHT [29] assemblies (data not shown) and manually curated to produce full mitochondrial genome assemblies. Single base ambiguities were resolved on a majority rules basis by remapping reads on regions of interest using bbmap v.38.84 (sourceforge.net/projects/bbmap/) and visualizing alignments in IGV v.2.8.2 [30]. Genomes were preliminarily annotated using Mitos [30] and manually curated based on known annotations of closely related species to produce final annotations. The new mitogenome sequences have been deposited in GenBank (under accession numbers MW238521 and MW238520), and the raw data that are deposited at the SRA available at the NCBI portal (accession number SRP289641).

### 2.3. Phylogenetic Analysis

All the available complete or semi-complete Collembolan mitogenomes were downloaded from GenBank in June 2020 together with those of three outgroups, *Daphnia pulex* (Crustacea, Branchiopoda), *Japyx solifugus* (Diplura, Japygidae) and *Trigoniophthalmus alternatus* (Microcoryphia, Machilidae). The sequences of *K. klovstadi* and *T. mixta*, produced in this study, were added to the final dataset, along with sequences from [20] curated as described by [2]. Available mitogenomes which lacked annotations (i.e., from [15]) were automatically annotated using Mitos [31]. The annotation of genomes displaying unusual features (long spacers, non-canonical gene order) were further revised manually. Genes that appeared truncated or totally different if compared to reference sequences were discarded.

Individual protein coding genes were extracted and retro-aligned in the RevTrans 2.0b server [32] based on an amino acid alignment produced using MAFFT v.7.309 [33]. Regions of unstable alignment were deleted with GBlocks v.0.91b [34] using the strict and codon options. Single gene alignments were concatenated to produce a final super matrix. Parallel analyses were conducted on the full matrix (i.e., all codon positions) and after removal of third codon positions. In both instances, data were divided in blocks by codon position, gene family and strand, as described by [2], and submitted to PartitionFinder v.2.1.1 [35] through the CIPRES Science Gateway [36] to determine the best partitioning scheme and associated evolutionary model. GTR+I+Γ was selected for all partitions except for the *atp8*, *nad6* and the first codon position of *nad5* and *nad4L*, for which the GTR+Γ was selected. Data matrices, as well as the optimal evolutionary model and partitioning scheme produced by PartitionFinder, were used for a MrBayes 3.2.6 [37] analysis using 8 chains and 100 million generations. The final output was summarized using tracer v.1.7.1 [38] excluding the first 25% as burn-in. The same datasets were used in a maximum likelihood analysis through IQ-TREE [39] with 10,000 iterations of ultra-fast bootstrap and evolutionary model optimized during the analysis. Final trees were visualized using Figtree v.1.4.4 [40].

### 2.4. Phylogenetic Tree Topology Comparison

To evaluate the support for some groups that, although well-defined taxonomically, were identified as polyphyletic in the analysis, constrained tree topologies were investigated using the RELL (Re-Estimated Log Likelihood) approximation method [41] with 10,000 replicates through IQ-TREE. Six different hypotheses were compared following [14]: (i) best unconstrained tree; (ii) monophyletic Neelipleona; (iii) monophyletic Tomoceridae; (iv) monophyletic Hypogastruridae; (v) monophyletic Paronellidae; (vi) monophyletic Entomobryidae.

### 2.5. Nucleotide Biases and dN/dS Ratio

Nucleotide biases were investigated on the same aligned data set. The matrix was divided into J-strand and N-strand oriented genes and, using an in-house python3 script, nucleotide skews were calculated as described in ref [42] (i.e., AT-skew = [A − T]/[A + T] and CG-skew = [C − G]/[C + G]), for first (J1 and N1), second (J2 and N2) and third codon positions of 2-fold-degenerate sites (2J3 and 2N3) and third codon position of 4-fold-degenerate sites (4J3 and 4N3). Outliers were defined as those sequences which showed a value beyond three times the interquartile range from the 25–75% quartile. A/T percentage was assessed on complete mtDNA sequences and compared with all Hexapoda mitochondrial genomes present in the organelle database (NCBI) in June 2020.

To check for positive or negative selection, the non-synonymous to synonymous ratio (ω = dN/dS) was evaluated on the same alignment after removal of outgroups. The alignment was processed as described above to obtain a phylogenetic tree that was used, in conjunction with the data, to test for selection in EasyCodeML [43] using the preset running mode with a site model setup. The dN/dS ratio was manually controlled for all models that showed a likelihood ratio test (LRT) with a significant *p*-value (≤ 0.5).

## 3. Results

### 3.1. New Mitogenomes

The mtDNA of *K. klovstadi* is a circular molecule of 15,486 bp length, and that of *T. mixta* is 14,998 bp length (see Tables S1 and S2 for details). Both genomes show the majority of genes encoded on the J-strand and display the typical features of metazoan mitochondrial genomes: 13 protein coding genes (*atp6*, *8*; *cox1*-3; *cytb*; *nad1-6*, *4L*), two rRNAs (*rrnL*, *rrnS*) and, in *K. klovstadi*, a complete set of 22 tRNAs. That of *T. mixta* lacks a *trnC*. This may represent a gene loss, although the possibility of a structurally divergent tRNA, that could not be recognized/annotated as such, cannot be ruled out completely. Canonical start codons (i.e., ATA/ATG, which encode for methionine) are present in the largest number of protein coding genes (8/13 in *T. mixta*, 6/13 in *K. klovstadi*). The alternative start codon TTG (leucine) is present in 1/13 protein coding genes (*nad4L*) in both mitogenomes, and ATT/ATC (isoleucine) are present in all other instances. Partial stop codons are frequent in *K. klovstadi* (9/13 protein coding genes) and present at lower frequencies (3/13) in *T. mixta*. Intergenic spacers are present in both genomes, with the longest being 592 bp (between *trnS* and *nad1*) in *K. klovsadi* and 155 bp (between *rrnL* and *trnV*) in *T. mixta*. Few overlaps between genes are present in both mtDNAs, and are limited to a small overlap between *atp6* and *atp8* in both genomes and a 33 bp overlap between *rrnL* and *trnV* in *K. klovstadi*. The gene arrangement for both species is consistent with the Pancrustacea model, except for *T. mixta* lacking *trnC*. Overall, a bias in nucleotide composition is observed toward A (36.2% and 34.0%) and T (31.6% and 29.1%), with respect to C (18.8% and 24.9%) and G (13.4% and 12.0%) in *K. klovstadi* and *T. mixta* mitogenomes, respectively.

### 3.2. Dataset Composition and Gene Orders

NCBI-sourced mitogenomes which lacked annotation were annotated as described in Section 2.3. In some cases, genomes were identified as incomplete due to the lack of some genes, sometimes associated to the presence of long stretches of Ns (undetermined nucleotides: 0.01% to 6.69%) in the original sequences.

**Figure 3:**
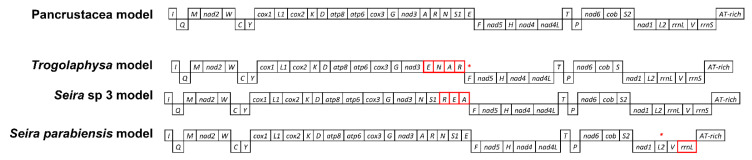
New gene order retrieved from the automatic annotation of [15] mitogenomic analysis compared to the ancestral Pancrustacean model. All derived gene arrangements, with respect to the basal model, show translocations as red boxes and deletions as asterisks (*).

The gene order was analyzed for all 87 species included in the study set with the exception of *Lepidocyrtus* sp. (MF716621), which displayed a highly divergent and uncertain structure and was therefore excluded (Tables S3 and S4). Most gene orders identified had been previously characterized and described [2,14,21,23]. Of the newly annotated genomes, *T. mixta* and *K. klovstadi* conform to the Pancrustacea model, with the exception of the missing *trnC* in the former (see Section 3.1). Most genomes from [15] similarly display a Pancrustacea gene order or, though incomplete, are compatible with it (*sensu* [2]; Table 1). A few species (*Seira ca. prodiga* 2, *S. downgli*, *S. tinguara*, *S. ritae*, *Entomobrya* sp. and *Tyrannoseira bielanensis*) have one or two missing genes. Nevertheless, in line with the observation that although no complete gene could be found/annotated, manual alignment with other genomes highlighted regions suggestive of homology, we cautiously did not assign a new gene order and tentatively consider these as conforming to the Pancrustacean gene order. A different gene order was observed in *Seira parabiensis*, characterized by an *rrnS* deletion and an *rrnL* translocation, while two species showed a genetic rearrangement in the *trnA-trnR-trnN-trnS-trnE* region. *Trogolaphysa* sp. (MF716607) displayed an inversion plus a deletion resulting in a *trnE-trnN-trnA-trnR* arrangement, and *Seira* sp. 3 (MF716612) showed a translocation resulting in a *trnN-trnS-trnR-trnE-trnA* configuration (Figure 3).

### 3.3. Phylogenetic Analysis

**Figure 4:**
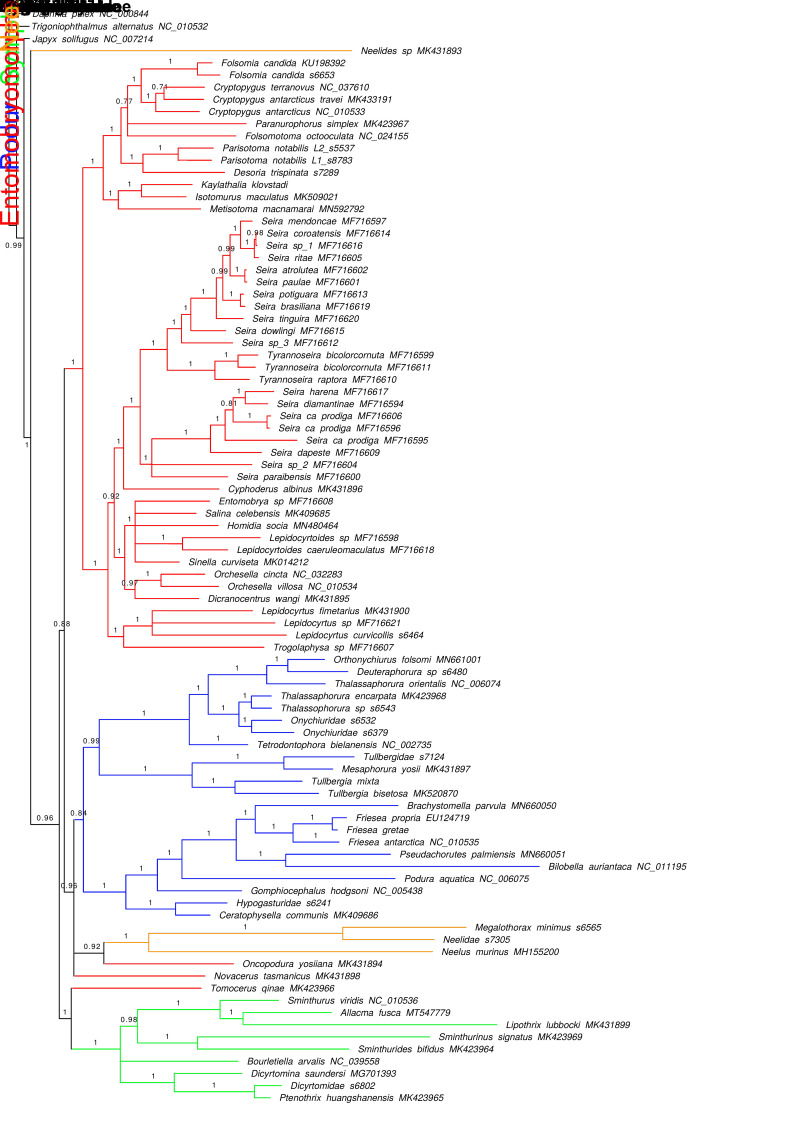
Bayesian phylogenetic reconstruction based on first and second codon positions. Numbers at nodes indicate posterior probabilities. Monophyletic, paraphyletic, and polyphyletic families are defined with filled, horizontal and vertical rods. Outliers species are highlighted with asterisks. Nodes with support < 0.70 were collapsed. Outgroup nodes not to scale.

The phylogenetic analysis of the dataset including first and second codon positions supports the monophyly of Collembola, as well as of the orders Symphypleona and Poduromorpha (Figure 4). The other two orders, Entomobryomorpha and Neelipleona, were identified to be polyphyletic due to the presence of outliers (*Neelides* sp., *Novacerus tasmanicus*, *Oncopodura yosiiana* and *Tomocerus qinae*), although the large majority of species formed two well-supported monophyletic clusters corresponding to the orders Entomobryomorpha and Neelipleona as taxonomically defined. At present, it is difficult to disentangle the possible causes of this outcome, whether it is a true polyphyly, a result of bias in the phylogenetic analyses or due to technical problems in the original data, although we suggest the former is the least likely.

One sequence, *Neelides* sp., is extremely divergent with respect to all other Collembolans and is recovered in the phylogenetic tree as the outermost basal group, distant from the other three representatives of Neelipleona that, in turn, form a tight monophyletic group nested in the Collembola tree. *Novacerus tasmanicus* and *Tomocerus qine* (belonging to the Tomoceridae subfamilies Lepidophorellinae and Tomocerinae, respectively) and *Oncopodura yosiiana* (subfamily Oncopodurinae) are all members of superfamily Tomoceroidea, but each cluster with different (and apparently unrelated) taxa (Figure 4).

At the level of orders, apart from the outliers, a well-supported and monophyletic Symphypleona branches at the basal position and is the sister group of the remaining lineages. These latter are subdivided into two major branches, the first including Poduromorpha and Neelipleona and the second including all Entomobryomorpha. Within Symphypleona, the represented families with two or more species (Sminthuridae and Dicyrtomidae) are recovered as monophyletic. At the family or subfamily level, Katiannidae is sister group to Sminthurididae, Sminthurinae is paraphyletic (*Allacma fusca* is more closely related to *Liphotrix lubbocki*, subfamily Sphyrothecinae, than to its con-subfamiliar genus *Sminthurus*). Subfamily Dicyrtominae (species *Dicyrtomina saundersi*) clusters together with Ptenothricinae (*Ptenothrix huangshanensis*). Entomobryomorpha, apart from the displacement of Oncopoduridea and Tomoceridae (the latter polyphyletic), form a coherent group. Isotomidae is monophyletic, whereas Entomobryidae and Paronellidae are polyphyletic. All Isotomidae genera, apart from *Sinella*, are monophyletic. Poduromorpha families are monophyletic with the exception of Hypogastruridae, due to the displacement of *Gomphiocephalus hodgsoni* as a sister taxon to Poduridae + Neanuridae. This monophyletic Poduromorpha is divided in two major clusters: one containing a paraphyletic Hypogastruridae (due to the displacement of *G. hodgsoni*) and Neanuridae, and the second including all Onychiuridae, the latter arranged as Tullbergiidae + (Tetrodontophorinae + Onychiurinae). In general, intermediate and recent nodes show full support, with minor exceptions, whereas nodes at the level of order and family are provided variable-to-high support (0.84 < p.p. < 1).

The tree resulting from the complete dataset (i.e., including third codon positions, Figure S1) is identical to that described above in terms of topology, with minor differences in support values. Maximum likelihood analysis produced a less resolved tree that, nevertheless, mostly conformed with the outcome of the Bayesian analyses (Figures S2 and S3). However, the collapsing of weakly supported branches (bootstrap value < 70) produced a large polytomy at the base of the tree and, with the exclusion of the aforementioned outliers, order monophyly was not confirmed only for one order (Poduromorpha) out of four, resulting from the splitting tree polytomy. Full support was detected for most other branches.

### 3.4. Tree Topology Tests

Monophyly tests conducted on ambiguous groups (i.e., taxonomically defined groups recovered as poly- or paraphyletic in the analysis) indicated that the competing hypothesis (i.e., monophyly) could not be rejected in all cases with full confidence. All non-monophyletic groups (Neelipleona, Tomoceridae, Hypogastruridae, Paronellidae and Entomobryidae) were therefore considered as topologically plausible, although suboptimal given our analysis (Table S5).

### 3.5. Nucleotide Biases and dN/dS Ratio

**Figure 5:**
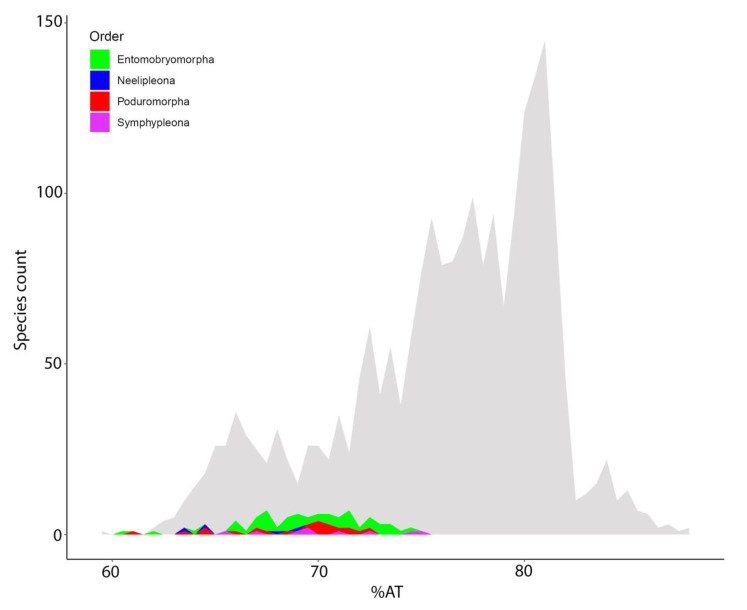
AT% bias within Hexapoda mitogenomes. Collembola genomes are color-coded against a grey background representing the entire Hexapoda dataset.

AT% composition of springtail mitogenomes was compared with data from all available hexapod mtDNAs. Although some internal variation was noted, Collembola appear to be characterized by a relatively low AT-bias (60.4–74.8%) compared to other Hexapoda (59.4–88%; Figure 5). 

**Figure 6:**
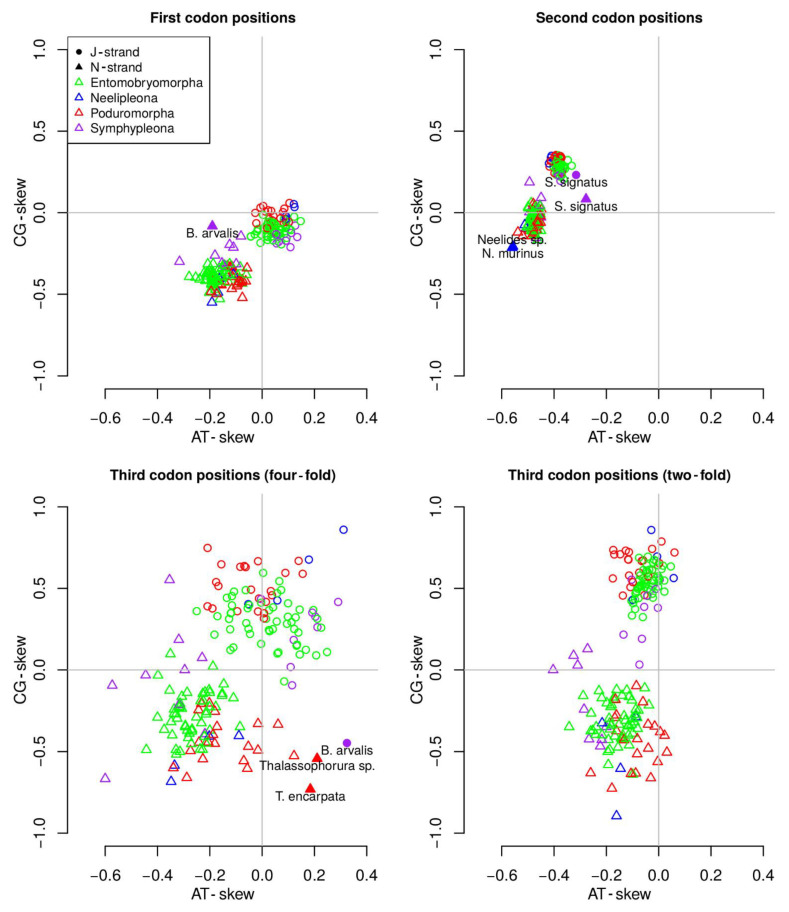
Single base skews for all available Collembola mitogenomes. Orders are color-coded and J- and N-strands are coded by shape. Outliers are identified by species name.

Nucleotide biases (AT skew and CG skew) were calculated for protein coding genes on both strands subdivided as first (J1 and N1), second (J2 and N2), two-fold (2J3 and 2N3) and four-fold (4J3 and 4N3) degenerate third codon positions. Variability in skew was sizeable in four-fold and two-fold third codon positions and limited in first and second codon positions, in the order 4JN3 > 2JN3 > JN2 > JN1 (Figure 6). All the Collembolan J1 genes showed a weak negative or barely positive CG skew (−0.21 to 0.06), whereas the AT skew was mainly positive (−0.04 to 0.15). N1 genes, on the other hand, showed strongly negative CG skew (−0.55 to −0.08) and AT skew (−0.32 to −0.06). The unique outlier for the N strand first nucleotide codon position bias was *Bourletiella arvalis*. Focusing on second codon positions, C outnumbered G in J2 position (0.19 to 0.35) but not in N2 positions (−0.21 to 0.19). AT skew at second codon positions was negative, with similar ranges for both strands (J2: −0.42 to −0.31; N2: −0.56 to −0.22). Two Neelipleona (*Neelides* sp. and *Neelus murinus*) and the Symphypleona *Sminthurinus signatus* differed in nucleotide composition from other Collembola species for the second nucleotide codon position. Third codon position biases displayed a much higher variability overall across species. Although AT skew for both 2J3 and 2N3 was comparable (−0.40 to 0.17), CG skew was particularly divergent, ranging from 0.03 to 0.86 in 2J3 and −0.89 to 0.2 in 2N3. No outlier was observed for the two-fold degenerate third codon position. Finally, 4N3 and 4J3 displayed the highest variance, with CG skews ranging from 0.85 to −0.73 and AT skews from 0.32 to −0.6. A substantial overlap was observed between values observed in the J and N strands, with the J strand being on the upper end of both values. Three outliers (*B. arvalis*, *Thalassophorura encarpata* and *Thalassophorura* sp.) were detected. 

The comparative models M0 vs M3, M7 vs M8 and M8a vs M8 led to a significant LRT *p*-value. However, the dN/dS ratio calculated for the nucleotide positions in all the Collembola species resulted in neutral selection, indicating that a balance for all the examined nucleotides was detected.

## 4. Discussion

Collembola phylogeny has long been a subject of debate, with different hypotheses stemming from the use of different markers (morphological and/or molecular) and the inclusion/exclusion of particular species. The mitochondrial genome has become an important resource in springtail systematics, but analyses have still suffered from limited taxon density and unequal representation of different lineages. In this study, we re-analyzed all available Collembola mitochondrial genomes in a unitary framework, currently the largest dataset of this type.

### 4.1. Deep Collembola Phylogeny

Analyses of the mitogenomes of the 87 species of Collembola represented in the dataset (Figure 4) produced phylogenetic trees that, although not completely resolved, were generally consistent, regardless of the use of different tree building methods (Bayesian Inference and ML) and different sets of data (including or excluding third codon positions). Overall, the four currently recognized orders were retrieved as monophyletic, with minor incongruences. Furthermore, tests conducted on constrained trees indicated that such incongruences (i.e., observed polyphyly in some taxa) were not strongly supported.

Comparison with previous studies highlights some clear dissimilarities, especially at deeper phylogenetic levels. The phylogenetic tree generated by ref 2 placed Neelipleona as the basal lineage of Collembola, with all orders monophyletic and with Symphypleona as a sister group to Entomobryomorpha [2]. Intra- and inter-familiar relationships generally conform to our study. Although Tullbergiidae was retrieved as sister taxon to all the other Poduromorpha families [2], in our analysis it formed a well-supported clade with Onychiuridae. A separate study concluded that Neelidae and Symphypleona, or Neelipleona alone, are basal to the remaining taxa, also finding general support for the monophyly of all orders (but not in all trees) [14]. The basal position of Neelipleona was also proposed using a set of nuclear markers [43]. Our tree has some similarity with that of ref 14, in suggesting a basal position for Symphypleona [14]. Notably, in our reconstruction, although three Neelipleona sequences (genera *Megalothorax* and *Neelus*, plus one undetermined specimen) clustered in a well-supported monophyletic group associated to Poduromorpha, one taxon (*Neelides* sp.) was placed in a basal position outside all other Collembola, as an outlier. We tentatively consider the former as the more likely placement for the Neelipleona. Moreover, Entomobryomorpha polyphyly and Hypogastruridae paraphyly, already reported by previous studies [2,11,14], conform to the current study’s analyses. Paronellidae and Entomobryidae, previously considered enigmatic taxa [44], were identified as polyphyletic as in Sun and colleagues’ phylogenetic reconstruction [14], casting doubt on the correct position of these groups.

The basal placement of Symphypleona, the Poduromorpha + Neelipleona clade, the close relationship between Isotomidae and (some) Entomobryidae, and the polyphyly of Entomobryomorpha due to the placement of Tomoceridae and Oncopoduridae, were also identified by [12] through the combined analysis of two conserved markers (*18S* and *28S*) in a dataset including 56 species. Polyphyly of Tomoceridae and Oncopoduridae was also recovered in one phylogenetic reconstruction by Sun et al. [14] in a study of more than 30 Collembola mitogenomes.

At a lower taxonomic level, in a previous study limited to the subfamily Seireinae and a few members of Entomobryidae [15], the genus *Seira* appeared to be paraphyletic due to the sister-group relationship between some species of this genus to those of *Tyrannoseira*. A similar outcome was obtained here, although we identified the Paronellidae species *Cyphoderus albinus* (instead of *Lepidocyrtus* sp.; missing in Nunes-Godeiro et al.’s dataset [15]) as the sister group of all *Seira* species.

### 4.2. Collembola Gene Orders

The gene order analysis confirmed that, in springtails, mitochondrial gene rearrangements are usually lineage-specific and represent autapomorphies or synapomorphic traits shared by low level taxonomic groups. The Pancrustacea model was confirmed as the ancestral gene order among Collembola due to its presence in all the studied orders, as well as in early divergent lineages above the family level. Nine other models have been observed in springtail mtDNAs (Table 1). The *Tetrodontophora* model is shared by all members of Onychiuridae and is suggested as a synapomorphy of the group. The first rearrangement of protein coding genes in springtails took place in Neanuridae, apparent in the *Pseudachorutes* model (Figure 2). No other species except *P. palminensis* show this mitochondrial gene order and, based on the resulting tree, we suggest that this feature is an autapomorphy of this group. If Symphypleona is the springtail order most likely to show genetic rearrangement, Entomobryomorpha is the opposite. Of the 52 genomes available for this group (of which the gene order of 51 was considered here), 49 displayed the ancestral Pancrustacea arrangement or, if incomplete, are compatible with this. The re-analysis of the mitogenomes described in ref 15 led to the identification of three new genomic models: *Trogolaphysa*, *Seira* sp. 3 and *Seira parabiensis* models [15]. Nevertheless, although their status as new gene orders is clearly plausible, caution is required because similar congeneric species (e.g., related to *Seira*) display the classical Pancrustacea gene order. Furthermore, although the first of these does not have other congeneric species that can be compared in terms of gene order, both *Seira* models are unique in the context of all other *Seira* species analyzed (19), that display the Pancrustacea model. 

The gene order analysis identified that the newly described genome of *T. mixta*, in contrast with the congeneric *T. bisetosa*, appears to lack *trnC*. The deletion of single tRNAs is not unprecedented either in mitochondrial genomes in general or Collembola specifically, but it is unusual, especially in congeneric species. Other possible explanations exist, such as that this species’ *trnC* assumed a variant morphology that is not captured by automatic annotation methods and/or by manual checking of the sequence. The possibility of an assembly error cannot be ruled out, nevertheless all evidence (high 2118 -fold coverage in the region between *trnW* and *trnY,* identical sequence produced by two independent assembly methods; see Section 2.2) suggest the opposite. Although, as noted, a result of automatic annotation, the only documented (to our knowledge) difference in genome structure identified in this study is the new *Seira* sp. 3 model, which differs from all the other congeneric species. 

Considering all the gene orders recognized in this study, it is clear that gene order modifications are not randomly distributed. Two genomic regions can be highlighted as being particularly subject to genetic modifications: the *A+T-rich*/*nad2* region, where most translocations were observed in Symphypleona; and the *trnA*/*nad1* region, that showed modifications in many taxa (Figure 2 and Figure 3). Symphypleona appear to be particularly prone to gene order modifications.

### 4.3. MtDNA Compositional Biases among Springtails

The replication of the mitochondrial genome is known to be an asynchronous and asymmetrical process, one outcome of which is that one (N-) hemi-helix remains in a single strand conformation for a prolonged period during replication (~two hours) and is therefore subject to ROS-induced directional mutations [41]. As described in previous studies, such a mutational bias is common in Collembola mitogenomes, which often show nucleotide compositional biases [21,45]. Comparison of the asymmetrical mutational constraints in all 87 species analyzed in this study led to some interesting findings. A+T bias, a well-known feature of insect mitochondrial genomes, was confirmed for all the analyzed Collembola, however they fall at the lower end of bias within Hexapoda [5,40] (Figure 5). Comparing codon positions, all springtails maintained a level of site-specific and strand-specific compositional conservation (Figure 6). In previous studies, it was generally observed that the J- and N-strand have tendencies to display an AC-rich and GT-rich base composition, respectively [14,45]. In the current study, GT-richness in the N-strand was confirmed, but AC-richness in the J-strand was less evident. This small, but noticeable, inconsistency among similar datasets could be partly due to the exclusion of hypervariable regions and to the fact that biases have been separately assessed for two- and four-fold degenerate sites. Being mt protein coding genes characterized by an abundance of hydrophobic amino acids (anchoring sites within the mitochondrial membrane systems), the second codon position is often occupied by pyrimidines (and especially thymine), whose presence fosters the encoding of hydrophobic amino acids. This type of bias is generally known as codon-bias, and all the Collembolan mitogenomes analyzed to date have demonstrated this feature [45]. 

Overall, different levels of dispersion were observed at different codon positions. First and second codon positions displayed the least dispersion, followed by two-fold and four-fold degenerate third codon positions. Given the degeneration of the genetic code, third codon position mutations rarely affect the sequence of the resulting protein and, hence, are more inclined to escape purifying selection than first and second codon positions [41]. Similarly, two-fold degenerate sites show lower mutational freedom than four-fold degenerate sites. Our observations are in line with expectations under this simple model of selection. In the context of a uniform distribution, some outliers were observed. The second codon position and four-fold degenerate sites in third codon position appear to include the majority of outliers both for the N- and J-strand genes. This may be the consequence of differential selection pressures in some specific taxa, but no clear pattern is apparent at present, although it is noticeable that most sequences detected as outliers belong to the same few taxa (*B. arvalis*, *S. signatus*, genus *Thalassophorura* and family Neelidae).

## 5. Conclusions

The evolutionary history of Collembola is a hotly debated topic among taxonomists and evolutionary biologists. In this study, we assembled, in a unitary framework, data analyzed in three mitogenomic studies, adding two new genomes and including manual curation of the data. This generated the largest dataset of complete or semi-complete Collembola mitochondrial genomes available to date. Differing from contemporary studies [2,14], we favored a basal position of Symphypleona and a close relationship between Poduromorpha and Neelipleona. However, suboptimal support for basal nodes, the misplacement of some taxa, and observation that, in the absence of a strong phylogenetic signal, the placement of taxa can be strongly influenced by taxon density, the molecular marker(s) applied, and the methods used, suggest caution before the phylogenetic relationships internal to the class Collembola can be considered as reliably assessed. 

Thanks to the technical developments in NGS sequencing of complete mitochondrial genomes, sequence data are accumulating rapidly. Nevertheless, although improved tools capable of unsupervised assembly and gene annotation are available, careful manual curation of the data remains important [46]. In this study, extensive manual curation of the data was used in order to include sequences that were not initially obtained as complete mitochondrial genomes, lacked annotations, or were present in NCBI outside the RefSeq area. This step led to the removal of some data and to the acceptance of other data as tentative. One of the major advantages of the use of complete mitochondrial sequences is the possibility to combine data across different studies and we, therefore, advocate that future sequencing studies strongly consider their reusability. Careful assembly and annotation, manual curation, post-annotation quality checks of the genome/annotation produced, and compliance to RefSeq standards should be seen at a shared necessity in this field when publishing complete mitochondrial genomes.

## Figures and Tables

**Table 1:** Species used in the current study. Gene order column asterisks (*) indicate a compatible assignment to the already described gene order. Section signs (^§^) indicate manual curated sequences originally obtained by [20]. Order names are abbreviated. Further information is available in Table S3. See GenBank for reference of downloaded sequences.

AN	Order	Family	Species	Gene Order
NC_010533	Entomobr.	Isotomidae	*Cryptopygus antarcticus*	Pancrustacea model
MK433191	Entomobr.	Isotomidae	*Cryptopygus a. travei*	Pancrustacea model
KX863671	Entomobr.	Isotomidae	*Cryptopygus terranovus*	Pancrustacea model
MK431896	Entomobr.	Paronellidae	*Cyphoderus albinus*	Pancrustacea model
s7289	Entomobr.	Isotomidae	*Desoria trispinata*	Pancrustacea model ^§^
MK431895	Entomobr.	Entomobryidae	*Dicranocentrus wangi*	Pancrustacea model
MF716608	Entomobr.	Entomobryidae	*Entomobrya* sp.	Pancrustacea model *
KU198392	Entomobr.	Isotomidae	*Folsomia candida*	Pancrustacea model
s6653	Entomobr.	Isotomidae	*Folsomia candida*	Pancrustacea model ^§^
NC_024155	Entomobr.	Isotomidae	*Folsomotoma octooculata*	Pancrustacea model
MN480464	Entomobr.	Entomobryidae	*Homidia socia*	Pancrustacea model
MK509021	Entomobr.	Isotomidae	*Isotomurus maculatus*	Pancrustacea model
MW238521	Entomobr.	Isotomidae	*Kaylathalia klovstadi*	Pancrustacea model
MF716618	Entomobr.	Entomobryidae	*Lepidocyrtoides caeruleomaculatus*	Pancrustacea model
MF716598	Entomobr.	Entomobryidae	*Lepidocyrtoides* sp.	Pancrustacea model
s6464	Entomobr.	Lepidocyrtinae	*Lepidocyrtus curvicollis*	Pancrustacea model ^§^
MK431900	Entomobr.	Entomobryidae	*Lepidocyrtus fimetarius*	Pancrustacea model
MF716621	Entomobr.	Lepidocyrtinae	*Lepidocyrtus* sp.	Not analysed
MN592792	Entomobr.	Isotomidae	*Metisotoma macnamarai*	Pancrustacea model
MK431898	Entomobr.	Tomoceridae	*Novacerus tasmanicus*	Pancrustacea model
MK431894	Entomobr.	Oncopoduridae	*Oncopodura yosiiana*	Pancrustacea model
KT985987	Entomobr.	Entomobryidae	*Orchesella cincta*	Pancrustacea model
EU016195	Entomobr.	Entomobryidae	*Orchesella villosa*	Pancrustacea model
MK423967	Entomobr.	Isotomidae	*Paranurophorus simplex*	Pancrustacea model *
s8783	Entomobr.	Isotomidae	*Parisotoma notabilis* L1	Pancrustacea model ^§^
s5537	Entomobr.	Isotomidae	*Parisotoma notabilis* L2	Pancrustacea model ^§^
MK409685	Entomobr.	Paronellidae	*Salina celebensis*	Pancrustacea model
MF716602	Entomobr.	Entomobryidae	*Seira atrolutea*	Pancrustacea model
MF716619	Entomobr.	Entomobryidae	*Seira brasiliana*	Pancrustacea model
MF716595	Entomobr.	Entomobryidae	*Seira ca. prodiga* 1	Pancrustacea model
MF716596	Entomobr.	Entomobryidae	*Seira ca. prodiga* 2	Pancrustacea model *
MF716606	Entomobr.	Entomobryidae	*Seira ca. prodiga* 3	Pancrustacea model
MF716614	Entomobr.	Entomobryidae	*Seira coroatensis*	Pancrustacea model
MF716609	Entomobr.	Entomobryidae	*Seira dapeste*	Pancrustacea model
MF716594	Entomobr.	Entomobryidae	*Seira diamantinae*	Pancrustacea model
MF716615	Entomobr.	Entomobryidae	*Seira dowlingi*	Pancrustacea model *
MF716617	Entomobr.	Entomobryidae	*Seira harena*	Pancrustacea model
MF716597	Entomobr.	Entomobryidae	*Seira mendoncae*	Pancrustacea model
MF716600	Entomobr.	Entomobryidae	*Seira paraibensis*	*Seira prarabiensis* model
MF716601	Entomobr.	Entomobryidae	*Seira paulae*	Pancrustacea model
MF716613	Entomobr.	Entomobryidae	*Seira potiguara*	Pancrustacea model
MF716605	Entomobr.	Entomobryidae	*Seira ritae*	Pancrustacea model *
MF716616	Entomobr.	Entomobryidae	*Seira* sp. 1	Pancrustacea model
MF716604	Entomobr.	Entomobryidae	*Seira* sp. 2	Pancrustacea model
MF716612	Entomobr.	Entomobryidae	*Seira* sp. 3	*Seira* sp. 3 model
MF716620	Entomobr.	Entomobryidae	*Seira tinguira*	Pancrustacea model *
MK014212	Entomobr.	Entomobryidae	*Sinella curviseta*	Pancrustacea model
MK423966	Entomobr.	Tomoceridae	*Tomocerus qinae*	Pancrustacea model
MF716607	Entomobr.	Paronellidae	*Trogolaphysa* sp.	*Trogolaphysa* model
MF716599	Entomobr.	Entomobryidae	*Tyrannoseira bicolorcornuta*	Pancrustacea model
MF716611	Entomobr.	Entomobryidae	*Tyrannoseira bicolorcornuta*	Pancrustacea model *
MF716610	Entomobr.	Entomobryidae	*Tyrannoseira raptora*	Pancrustacea model
s7305	Neeliple.	Neelidae	gen. sp.	Pancrustacea model ^§^
s6565	Neeliple.	Neelidae	*Megalothorax minimus*	Pancrustacea model ^§^
MK431893	Neeliple.	Neelidae	*Neelides* sp.	Pancrustacea model *
MH155200	Neeliple.	Neelidae	*Neelus murinus*	Pancrustacea model
EU084034	Podurom.	Neanuridae	*Bilobella aurantiaca*	Pancrustacea model
MN660050	Podurom.	Brachystomellidae	*Brachystomella parvula*	Pancrustacea model *
MK409686	Podurom.	Hypogastruridae	*Ceratophysella communis*	Pancrustacea model
s6480	Podurom.	Onychiuridae	*Deuteraphorura* sp.	*Tetrodontophora* model ^§^
NC010535	Podurom.	Neanuridae	*Friesea antarctica*	Pancrustacea model
MT644085	Podurom.	Neanuridae	*Friesea gretae*	Pancrustacea model
EU124719	Podurom.	Neanuridae	*Friesea propria*	Pancrustacea model
s6241	Podurom.	Hypogastruridae	gen. sp.	Pancrustacea model ^§^
s6379	Podurom.	Onychiuridae	gen. sp.	*Tetrodontophora* model ^§^
s6532	Podurom.	Onychiuridae	gen. sp.	*Tetrodontophora* model^§^
s7124	Podurom.	Tullbergiidae	gen. sp.	Pancrustacea model *^§^
AY191995	Podurom.	Hypogastruridae	*Gomphiocephalus hodgsoni*	Pancrustacea model
MK431897	Podurom.	Tullbergiidae	*Mesaphorura yosii*	Pancrustacea model
MN661001	Podurom.	Onychiuridae	*Orthonychiurus folsomi*	*Tetrodontophora* model
NC_006075	Podurom.	Poduridae	*Podura aquatica*	*Podura* model *
MN660051	Podurom.	Neanuridae	*Pseudachorutes palmiensis*	*Pseudachorutes* model
NC_002735	Podurom.	Onychiuridae	*Tetrodontophora bielanensis*	*Tetrodontophora* model
MK423968	Podurom.	Onychiuridae	*Thalassaphorura encarpata*	*Tetrodontophora* model
NC_006074	Podurom.	Onychiuridae	*Thalassaphorura orientalis*	*Tetrodontophora* model *
s6543	Podurom.	Onychiuridae	*Thalassophorura* sp.	*Tetrodontophora* model ^§^
MK520870	Podurom.	Tullbergiidae	*Tullbergia bisetosa*	Pancrustacea model
MW238520	Podurom.	Tullbergiidae	*Tullbergia mixta*	Pancrustacea model *
MT547779	Symphy.	Sminthuridae	*Allacma fusca*	*Allacma* model
KY618680	Symphy.	Bourletiellidae	*Bourletiella arvalis*	Pancrustacea model
MG701393	Symphy.	Dicyrtomidae	*Dicyrtomina saundersi*	Pancrustacea model
s6802	Symphy.	Dicyrtomidae	gen. sp.	Pancrustacea model ^§^
MK431899	Symphy.	Sminthuridae	*Lipothrix lubbocki*	*Lipothirx* model
MK423965	Symphy.	Dicyrtomidae	*Ptenothrix huangshanensis*	Pancrustacea model
MK423964	Symphy.	Sminthurididae	*Sminthurides bifidus*	Pancrustacea model *
MK423969	Symphy.	Katiannidae	*Sminthurinus signatus*	*Sminthurinus* model *
NC_010536	Symphy.	Sminthuridae	*Sminthurus viridis*	*Sminthurus* model

## Data Availability

The new data presented in this study are openly available in GenBank of NCBI at https://www.ncbi.nlm.nih.gov/nuccore/MW238520 and https://www.ncbi.nlm.nih.gov/nuccore/MW238521, with reference numbers MW238520 and MW238521.

## Supplementary Materials

The following are available online at https://www.mdpi.com/2073-4425/12/1/44/s1, Figure S1: Bayesian Inferenced phylogenetic Figure S2: Maximum likelihood phylogenetic tree (2 codon position), Figure S3: Maximum likelihood phylogenetic tree (3 codon position), Table S1 Genome annotation of *Kaylathalia klovstadi*, Table S2: Genome annotation of *Tullbergia mixta*, Table S3: Taxa under study, detail of authority, sampling location, genes and mitogenome length, Table S4: New genome automatic annotation, Table S5: Tree topology tests across non monophyletic groups of Collembola.
