# Supplementary material for: Re-Evaluating the Internal Phylogenetic Relationships of Collembola by Means of Mitogenome Data
Source: Genes (Basel). 2020 Dec 30;12(1):44. doi: 10.3390/genes12010044 (PMC7824276; doi:10.3390/genes12010044)
Supplement: Supplementary file 1 [file genes-12-00044-s001.zip › SM/Table S1 Genome annotation of Kaylathalia klovstadi.docx]

| Gene | A% | C% | G% | T% | Length (bp) | Strand | Position | Spacers/overlaps | Start codon | Stop codon |
| --- | --- | --- | --- | --- | --- | --- | --- | --- | --- | --- |
| *trnI* | 34.8 | 9.1 | 15.2 | 40.9 | 66 | J | 1-66 |  |  |  |
| *trnQ* | 39.7 | 13.2 | 7.4 | 39.7 | 68 | N | 133-66 | -1 |  |  |
| *trnM* | 26.1 | 26.1 | 18.8 | 29.0 | 69 | J | 133-201 | -1 |  |  |
| *nad2* | 29.2 | 19.9 | 14.5 | 36.4 | 995 | J | 203-1197 | 1 | ATT (I) | TA- |
| *trnW* | 37.3 | 13.4 | 13.4 | 35.8 | 67 | J | 1198-1264 | 0 |  |  |
| *trnC* | 29.5 | 21.3 | 14.8 | 34.4 | 61 | N | 1329-1269 | 4 |  |  |
| *trnY* | 30.6 | 27.4 | 16.1 | 25.8 | 62 | N | 1391-1330 | 0 |  |  |
| *cox1* | 27.8 | 20.8 | 18.1 | 33.2 | 1534 | J | 1392-2925 | 0 | TTG (L) | T-- |
| *trnL2* | 36.9 | 10.8 | 18.5 | 33.8 | 65 | J | 2926-2990 | 0 |  |  |
| *cox2* | 31.4 | 21.3 | 14.0 | 33.3 | 685 | J | 2991-3675 | 0 | ATA (M) | T-- |
| *trnK* | 35.2 | 22.5 | 15.5 | 26.8 | 71 | J | 3676-3746 | 0 |  |  |
| *trnD* | 38.7 | 8.1 | 12.9 | 40.3 | 62 | J | 3746-3807 | -1 |  |  |
| *atp8* | 35.8 | 20.4 | 13.6 | 30.2 | 162 | J | 3808-3969 | 0 | ATA (M) | TAA |
| *atp6* | 28.3 | 20.0 | 13.7 | 38.0 | 681 | J | 3963-4643 | -7 | ATG (M) | TAA |
| *cox3* | 25.5 | 20.7 | 18.1 | 35.7 | 784 | J | 4646-5429 | 2 | ATA (M) | T-- |
| *trnG* | 38.7 | 12.9 | 12.9 | 35.5 | 62 | J | 5430-5491 | 0 |  |  |
| *nad3* | 30.9 | 23.3 | 14.3 | 31.5 | 343 | J | 5492-5834 | 0 | ATT (I) | T-- |
| *trnA* | 31.2 | 12.5 | 20.3 | 35.9 | 64 | J | 5835-5898 | 0 |  |  |
| *trnR* | 29.5 | 16.4 | 16.4 | 37.7 | 61 | J | 5898-5958 | -1 |  |  |
| *trnN* | 35.9 | 14.1 | 20.3 | 29.7 | 64 | J | 5957-6020 | -2 |  |  |
| *trnS1* | 35.8 | 13.4 | 13.4 | 37.3 | 67 | J | 6019-6085 | -2 |  |  |
| *trnE* | 37.5 | 10.9 | 7.8 | 43.8 | 64 | J | 6087-6150 | 1 |  |  |
| *trnF* | 26.6 | 34.4 | 17.2 | 21.9 | 64 | N | 6215-6152 | 1 |  |  |
| *nad5* | 43.7 | 18.5 | 12.1 | 25.7 | 1702 | N | 7917-6216 | 0 | ATC (I) | T-- |
| *trnH* | 37.1 | 22.6 | 9.7 | 30.6 | 62 | N | 7979-7918 | 0 |  |  |
| *nad4* | 44.7 | 19.9 | 11.9 | 23.5 | 1362 | N | 9341-7980 | 0 | ATG (M) | TAG |
| *nad4l* | 50.0 | 16.5 | 10.4 | 23.0 | 278 | N | 9619-9342 | 0 | TTG (L) | TA- |
| *trnT* | 38.7 | 12.9 | 14.5 | 33.9 | 62 | J | 9622-9683 | 2 |  |  |
| *trnP* | 41.0 | 23.0 | 9.8 | 26.2 | 61 | N | 9744-9684 | 0 |  |  |
| *nad6* | 34.6 | 17.4 | 12.3 | 35.7 | 488 | J | 9750-10237 | 5 | ATT (I) | TA- |
| *cob* | 29.7 | 20.3 | 16.3 | 33.7 | 1133 | J | 10238-11370 | 0 | ATG (M) | TA- |
| *trnS2* | 32.4 | 16.9 | 16.9 | 33.8 | 71 | J | 11371-11441 | 0 |  |  |
| *nad1* | 45.2 | 19.5 | 12.3 | 22.9 | 942 | N | 12975-12034 | 592 | ATT (I) | TAA |
| *trnL1* | 38.1 | 17.5 | 12.7 | 31.7 | 63 | N | 13041-12979 | 3 |  |  |
| *rrnL* | 40.5 | 17.3 | 10.4 | 31.8 | 1248 | N | 14289-13042 | 0 |  |  |
| *trnV* | 34.9 | 20.6 | 19.0 | 25.4 | 63 | N | 14319-14257 | -33 |  |  |
| *rrnS* | 39.2 | 17.4 | 10.5 | 32.9 | 696 | N | 15011-14316 | -4 |  |  |
| A+T-ric | 43.0 | 5.7 | 4.4 | 46.8 | 474 | J | 15012-15485 | 0 |  |  |
| total | 36.1 | 18.8 | 13.5 | 31.6 | 15485 |  |  |  |  |  |

Table S1 Annotation of *Kaylathalia klovstadi* mitogenome. Nucleotide composition is calculated on the J strand.
