# Supplementary material for: Re-Evaluating the Internal Phylogenetic Relationships of Collembola by Means of Mitogenome Data
Source: Genes (Basel). 2020 Dec 30;12(1):44. doi: 10.3390/genes12010044 (PMC7824276; doi:10.3390/genes12010044)
Supplement: Supplementary file 1 [file genes-12-00044-s001.zip › SM/Table S2 Genome annotation of Tullbergia mixta.docx]

| Gene | A% | C% | G% | T% | Length (bp) | Strand | Position | Spacers/overlaps | Start codon | Stop codon |
| --- | --- | --- | --- | --- | --- | --- | --- | --- | --- | --- |
| *trnI* | 30.2 | 15.9 | 22.2 | 31.7 | 63 | J | 1-63 |  |  |  |
| *trnQ* | 38.8 | 23.9 | 6.0 | 31.3 | 67 | N | 132-66 | 2 |  |  |
| *trnM* | 30.2 | 25.4 | 15.9 | 28.6 | 63 | J | 132-194 | -1 |  |  |
| *nad2* | 27.6 | 27.6 | 10.6 | 34.2 | 987 | J | 216-1202 | 21 | ATA (M) | TAA |
| *trnW* | 41.2 | 16.2 | 14.7 | 27.9 | 68 | J | 1201-1268 | -2 |  |  |
| *trnY* | 41.0 | 23.0 | 9.8 | 26.2 | 61 | N | 1389-1329 | 60 |  |  |
| *cox1* | 26.9 | 25.3 | 15.9 | 31.9 | 1527 | J | 1400-2926 | 10 | ATA (M) | TAA |
| *trnL2* | 39.7 | 14.3 | 15.9 | 30.2 | 63 | J | 2933-2995 | 6 |  |  |
| *cox2* | 30.8 | 27.3 | 12.7 | 29.1 | 684 | J | 2996-3679 | 0 | ATT (I) | TAA |
| *trnK* | 39.1 | 13.0 | 15.9 | 31.9 | 69 | J | 3682-3750 | 2 |  |  |
| *trnD* | 38.7 | 16.1 | 17.7 | 27.4 | 62 | J | 3751-3812 | 0 |  |  |
| *atp8* | 28.6 | 29.8 | 12.5 | 29.2 | 168 | J | 3813-3980 | 0 | ATT (I) | TAA |
| *atp6* | 26.5 | 27.9 | 13.7 | 31.9 | 678 | J | 3977-4654 | -4 | ATA (M) | TAA |
| *cox3* | 26.6 | 25.8 | 15.6 | 31.9 | 786 | J | 4675-5460 | 20 | ATA (M) | TAA |
| *trnG* | 33.3 | 21.7 | 20.0 | 25.0 | 60 | J | 5463-5522 | 2 |  |  |
| *nad3* | 23.5 | 31.0 | 13.3 | 32.2 | 345 | J | 5523-5867 | 0 | ATC (I) | TAA |
| *trnA* | 31.7 | 15.0 | 20.0 | 33.3 | 60 | J | 5873-5932 | 5 |  |  |
| *trnR* | 26.8 | 28.6 | 17.9 | 26.8 | 56 | J | 5937-5992 | 4 |  |  |
| *trnN* | 37.7 | 13.1 | 19.7 | 29.5 | 61 | J | 5993-6053 | 0 |  |  |
| *trnS1* | 23.3 | 23.3 | 18.3 | 35.0 | 60 | J | 6054-6113 | 0 |  |  |
| *trnE* | 39.7 | 15.9 | 14.3 | 30.2 | 63 | J | 6115-6177 | 1 |  |  |
| *trnF* | 36.5 | 22.2 | 9.5 | 31.7 | 63 | N | 6240-6178 | 0 |  |  |
| *nad5* | 42.3 | 25.2 | 11.7 | 20.9 | 1708 | N | 7948-6241 | 0 | ATA (M) | T-- |
| *trnH* | 38.3 | 20.0 | 6.7 | 35.0 | 60 | N | 8008-7949 | 0 |  |  |
| *nad4* | 42.6 | 25.9 | 10.8 | 20.8 | 1348 | N | 9377-8030 | 21 | ATG (M) | T-- |
| *nad4l* | 47.8 | 25.9 | 7.4 | 18.9 | 270 | N | 9649-9380 | 2 | TTG (L) | TAA |
| *trnT* | 32.3 | 16.9 | 13.8 | 36.9 | 65 | J | 9660-9724 | 10 |  |  |
| *trnP* | 37.7 | 21.3 | 11.5 | 29.5 | 61 | N | 9782-9722 | -3 |  |  |
| *nad6* | 29.7 | 24.3 | 10.1 | 35.9 | 474 | J | 9787-10260 | 4 | ATA (M) | TAA |
| *cob* | 28.8 | 26.2 | 13.5 | 31.5 | 1135 | J | 10268-11402 | 7 | ATG (M) | T-- |
| *trnS2* | 35.7 | 12.9 | 14.3 | 37.1 | 70 | J | 11403-11472 | 0 |  |  |
| *nad1* | 43.0 | 25.2 | 10.3 | 21.5 | 924 | N | 12542-11619 | 146 | ATT (I) | TAG |
| *trnL1* | 37.7 | 16.4 | 8.2 | 37.7 | 61 | N | 12644-12584 | 41 |  |  |
| *rrnL* | 36.5 | 23.0 | 9.2 | 31.4 | 993 | N | 13677-12685 | 40 |  |  |
| *trnV* | 29.9 | 25.4 | 14.9 | 29.9 | 67 | N | 13899-13833 | 155 |  |  |
| *rrnS* | 34.8 | 23.1 | 9.5 | 32.5 | 726 | N | 14623-13898 | -2 |  |  |
| A+T-rich | 36.0 | 12.8 | 8.0 | 43.2 | 375 | J | 14624-14998 | 0 |  |  |
| total | 34.0 | 24.9 | 12.0 | 29.1 | 14998 |  |  |  |  |  |

Table S2. Annotation of *Tullbergia* *mixta* mitogenome. Nucleotide composition is calculated on the J strand.
