# Supplementary figures and images for: Re-Evaluating the Internal Phylogenetic Relationships of Collembola by Means of Mitogenome Data
Source: Genes (Basel). 2020 Dec 30;12(1):44. doi: 10.3390/genes12010044 (PMC7824276; doi:10.3390/genes12010044)

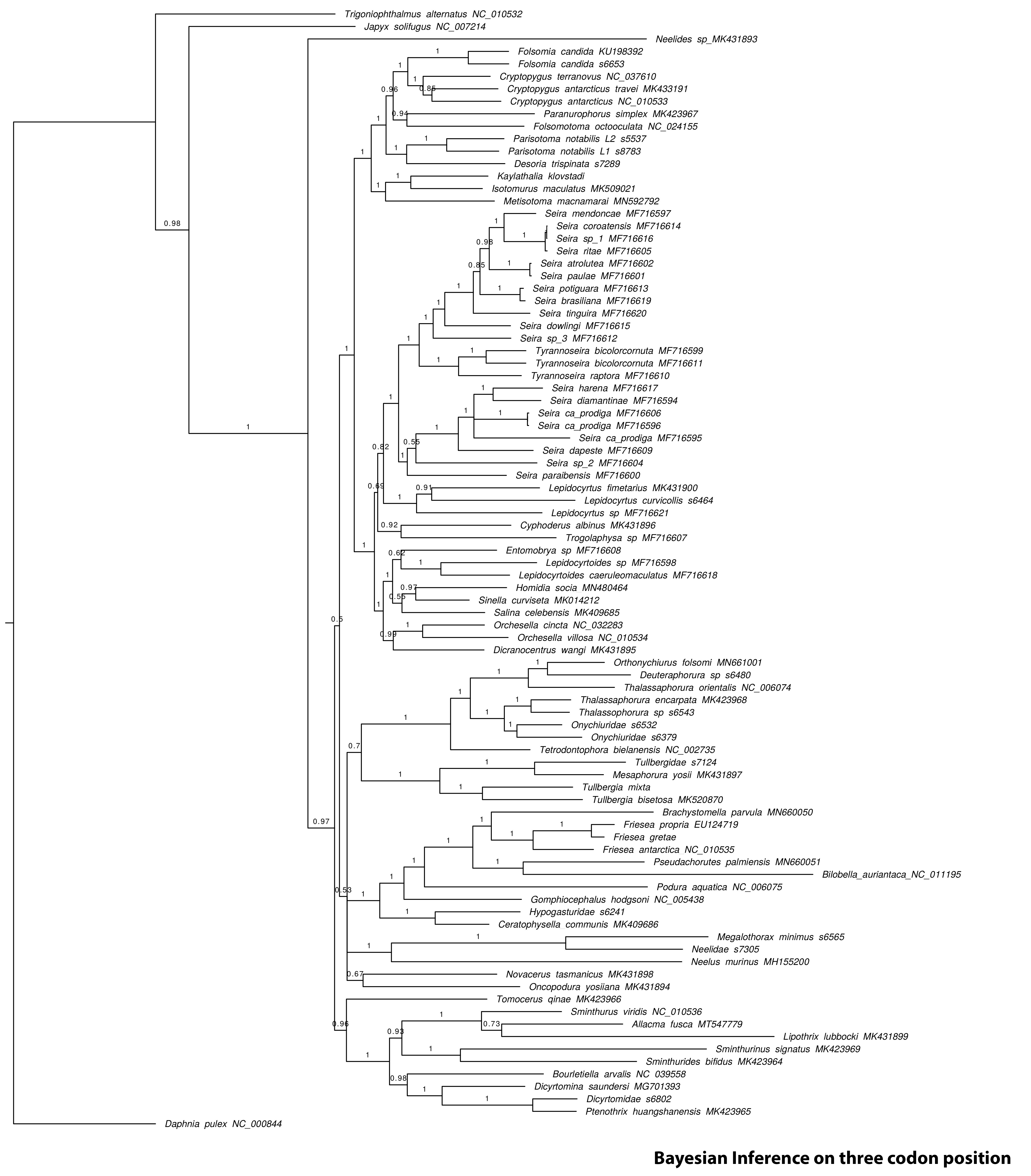

Supplement: Supplementary file 1 [file genes-12-00044-s001.zip › SM/Figure S1 Bayesian Inferenced phylogenetic tree (3 codon position).jpg]

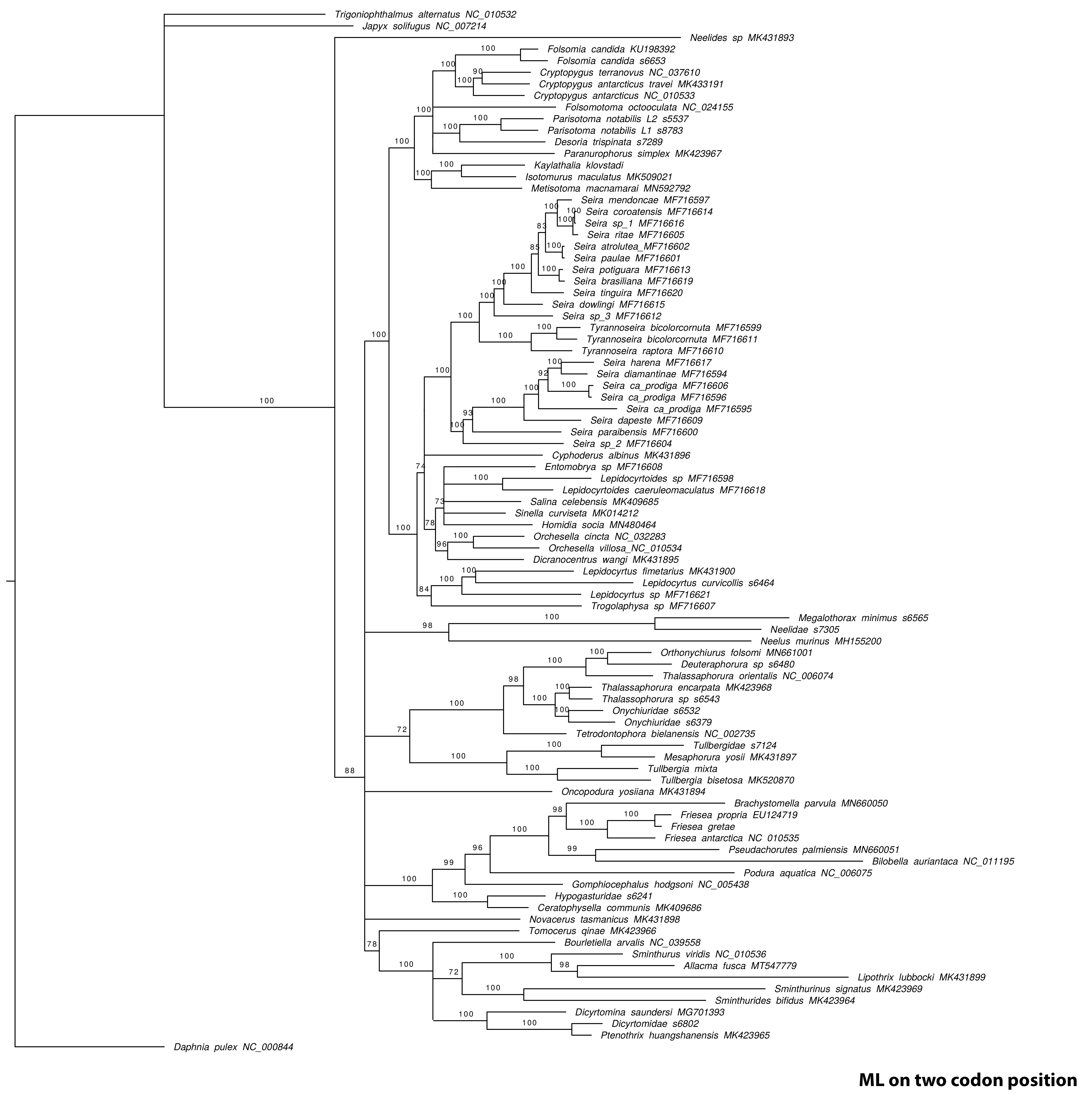

Supplement: Supplementary file 1 [file genes-12-00044-s001.zip › SM/Figure S2 Maximum Likelihood phylogenetic tree (2 codon position).jpg]

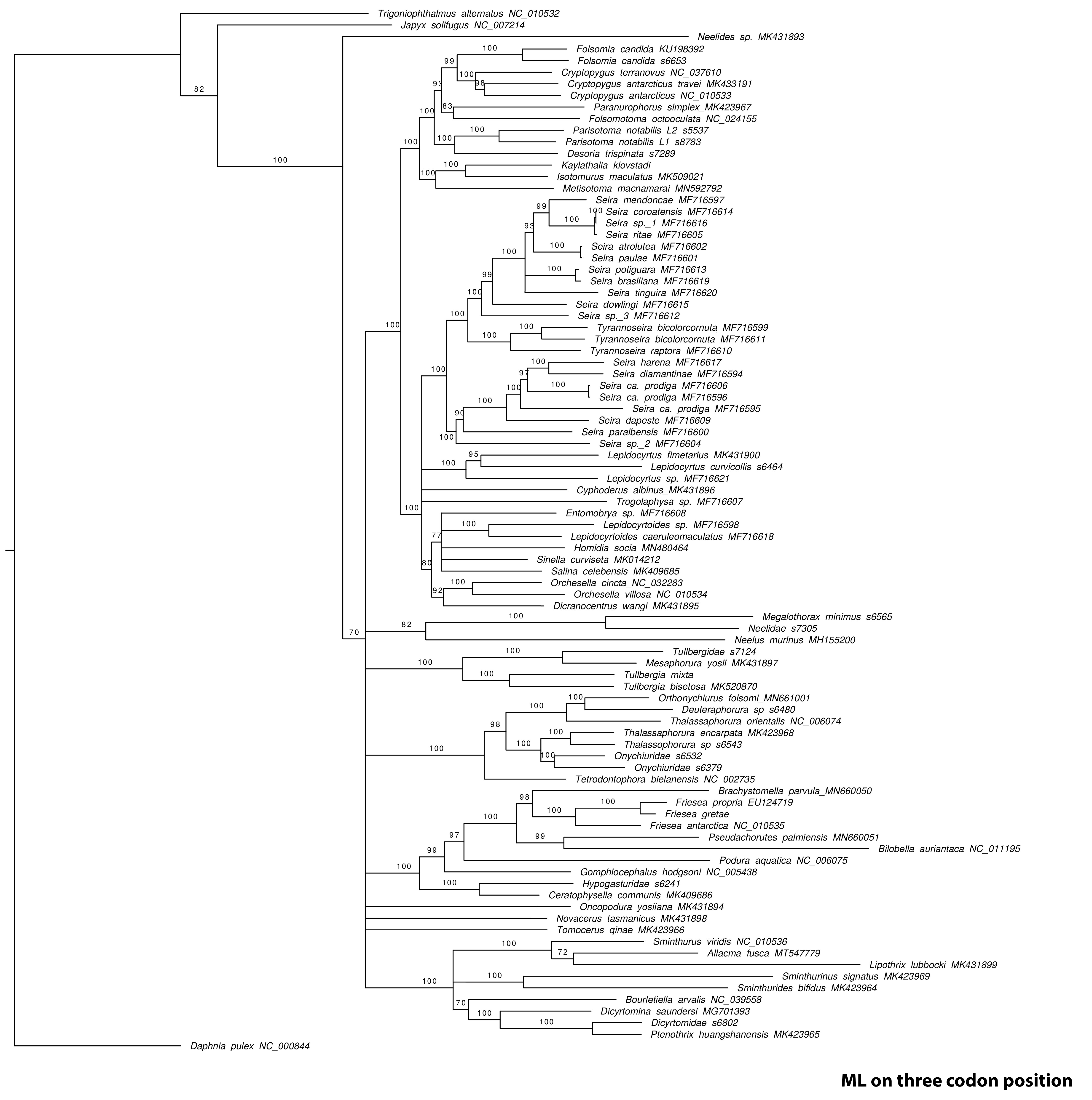

Supplement: Supplementary file 1 [file genes-12-00044-s001.zip › SM/Figure S3 Maximum Likelihood phylogenetic tree (3 codon position).jpg]
